# Annual Meeting of the Illinois State Medical Society

**Published:** 1857-04

**Authors:** 


*Annual Meeting of the Illinois State Medical Society.*

The next annual meeting of this Society will be held in the city of Chicago, commencing on the first Tuesday in June next. We trust that all the local societies in the State will bear this in mind, and appoint such delegates as will attend. We would remind members of the profession, residing in counties where no local societies exist, that if they attend, with evidence of their being graduates and in good standing, they will be cordially elected as permanent members and be allowed to participate in the business of the meeting. We confidently anticipate a full and profitable meeting of the Society. The following is the Committee of Arrangements, viz:—Drs. J. H. Hollister, De Laskie Miller, J. Bloodgood, C. G. Smith, and Henry Parker.

Dr. J. V. Z. Blaney, is also Chairman of the Committee on Prize Essays; and all papers intended for that purpose, should be placed in his hands on or before the first day of May, 1857.

